# Slave Trade and Hepatitis B Virus Genotypes and Subgenotypes in Haiti and Africa

**DOI:** 10.3201/eid1508.081642

**Published:** 2009-08

**Authors:** Iris E. Andernach, Claudine Nolte, Jean W. Pape, Claude P. Muller

**Affiliations:** Author affiliations: Institute of Immunology, Luxembourg, Luxembourg (I.E. Andernach, C.P. Muller);; Groupe d’Etude du Sarcome de Kaposi et des Infections Opportunistes, Port-au-Prince, Haiti (C. Nolte, J.W. Pape);; Cornell University, Ithaca, New York, USA (J.W. Pape).

**Keywords:** Hepatitis B virus, genotype, Haiti, Caribbean, Africa, viruses, transatlantic slave trade, prevalence, research

## Abstract

In Haiti, >90% of the population descended from African slaves. Of 7,147 Haitian pregnant women sampled, 44% of hepatitis B virus (HBV) infections were caused by genotype A1, which today is found mainly in eastern Africa. Twenty percent belong to a rare subgenotype, A5, which has been found only in the former Bight of Benin, a former primary slave trading post. Haitian A subgenotypes appear to have separated early from the African subgenotypes; the most prevalent genotype and subgenotype in West Africa today (E and A3, respectively) are rare in Haiti. This difference indicates that the dominant subgenotypes in Africa emerged in the general population only after the slave trade and explains the low genetic diversity of genotype E. The high prevalence of HBV genotype E in much of Africa further suggests that HBV hyperendemicity is a recent phenomenon, probably resulting from extensive use of unsafe needles.

## Abstract 2

TOC Summary: The spread of genotype E in Africa occurred after the end of the transatlantic slave trade.

Because of a viral polymerase that lacks proofreading activity (*1*), hepatitis B virus (HBV) has evolved into at least 8 recognized genotypes, A–H (*2*–*4*), and a potential new genotype (tentatively designated genotype I) found mainly in Laos (*5*,*6*) but also in Vietnam (*7*). Except for genotypes E, G, and H, genotypes can be further divided into a variety of subgenotypes, sometimes with more or less geographic distribution. Genotype D strains are found almost worldwide (*8*), but subgenotype D1 occurs mostly in the Mediterranean and Middle East but also in Europe. D2 has been reported in India, Japan, Europe, and the United States; D3, mainly in South Africa and Brazil but also in Rwanda, Costa Rica, the United States, and Europe (*8*–*11*); and D4, in Australia, South Africa, Somalia, Rwanda, and Oceania (*8*–*10*).

In sub-Saharan Africa, genotypes E and A predominate. East of the E/A divide (*9*), subgenotype A1 is dominant in countries along the eastern coast from South Africa to the Horn of Africa (*12*). Although genotype A has been found on every continent, its genetic diversity is higher in Africa (4% over the complete genome) than in the rest of the world (3%). Five subtypes of HBV/A (A1–A5) have been proposed in Africa (*13*), whereas essentially only A2 and, to a lesser extent, A1 have been reported from other continents (*14*). Therefore, some researchers have suggested that genotype A has emerged in Africa (*15*) and, after a long evolution, has been introduced to other continents. However, despite the high genetic diversity of HBV/A in West Africa, this genotype is rare there. In contrast, genotype E has been found only in Africa, with some rare exceptions on other continents in persons with a link to Africans. Genotype E is found almost exclusively throughout the vast expanses of a crescent from Senegal in the west (*16*) to the Central African Republic in the east (*17*) and Namibia in the south (*13*). In comparison to HBV/A, the conspicuously low genetic diversity of HBV/E suggests its short natural history in Africa (*18*) and relatively recent introduction into the general population there (*18*). However, the recent presence of HBV/E in Africa contrasts sharply with its current high prevalence and extensive geographic distribution there. The wide spread of genotype E also seems difficult to reconcile with a long natural history of genotype A in Africa (*18*).

In Haiti, where >90% of the population descends directly from African slaves (*19*), we investigated the phylogeny of HBV to learn which genotypes may have been prevalent in Africa several centuries ago. The conspicuous absence of genotype E in Haiti suggests recent and rapid spread of genotype E in Africa during the past 200 years, probably as the result of public health interventions.

## Materials and Methods

Serum samples were collected in 2006 after informed consent as part of a national survey to evaluate prevalences of human immunodeficiency virus infection, hepatitis B, and serologic syphilis among pregnant women at their first prenatal medical visit in 19 clinics throughout Haiti. Women were tested for hepatitis B surface antigen (HBsAg) by using the Murex HBsAg Kit (Abbott Laboratories, Ottiginies, Belgium). DNA was extracted from HBsAg–positive samples by using the QIAGEN DNA Blood Mini kit (QIAGEN, Venlo, the Netherlands) according to the manufacturer’s protocol. The complete HBV genome was amplified in 4 overlapping fragments (preS, S, X, and C) as described previously (*20*). Phylogenetic analysis and distance calculations were performed by using MEGA v.4 (*21*) with the neighbor-joining method of the Kimura 2-parameter model with 1,000 bootstrap replicates. Genotyping was performed by analyzing the complete genome or at least 1 of the 3 fragments of preS, S, or C genes. Subgenotyping was done on the full-length genome or on at least 2 complete fragments preS, S, or C. Sequences were submitted to EMBL/GenBank/DDBJ under accession nos. FJ692502–FJ692553 (Haiti S-fragment sequences), FJ692557–FJ692613 (Haiti complete genome sequences), and FJ692554–FJ692556 (Nigeria complete A5 sequences).

## Results

### Genotypes and Subgenotypes

In 7,147 blood samples of pregnant Haitian women, HBsAg prevalence was 5%, ranging from 1.0% to 8.5%, depending on the sampling clinic. Of 320 HBsAg–positive samples available, 247 (77.2%) were positive for at least 1 of the 4 overlapping PCR fragments (Table 1). Interpretable sequences from at least 1 of the 4 PCR fragments were obtained from 213 viruses. A total of 179 of these strains could be clearly assigned to a genotype by analyzing the complete genome or at least 1 of the 3 fragments of preS, S, or C genes. Of the 213 strains, 31 showed signs of mixed infection or recombination, and 3 strains were considered outliers because they could not be genotyped.

**Table 1:** Number of serum samples investigated, including suspected mixed and recombinant strains of HBV, Haiti*

Samples	No. samples (no. partial sequences)
No. serum samples (HBsAg positive) available	320
No. serum samples PCR positive	247
No. serum samples for which sequences were obtained†	182
Full-length genome	68
Full preS fragment	57 (37)
Full S fragment	67 (19)
Full X fragment	67 (20)
Full C fragment	20 (34)
No. serum samples of suspected mixed strains	25
Full-length genome	10
Full preS fragment	8 (3)
Full S fragment	12 (2)
Full X fragment	10 (2)
Full C fragment	3 (3)
No. serum samples of suspected recombinant strains	6
preS fragment	3
S fragment	–
X fragment	3
C fragment	–

*HBV, hepatitis B virus; HBsAg, hepatitis B surface antigen. †No. serum samples includes 3 outliers and excludes mixed or recombinant strains.

Phylogenetic analysis of the above 179 genotypeable strains (excluding mixed, recombinant, and untypeable strains) showed that 128 (71.5%) viruses belonged to genotype A; 40 (22.4%), to genotype D; and 11 (6.1%), to genotype E (Table 2). Genotype A strains were attributable to subgenotypes A1 (n = 77 [43.0%]) and A5 (n = 35 [19.6%]). Genotype D strains belonged to D4 (16.2%) and D3 (3.9%). Fifteen viruses of genotype A and 4 of genotype D could not be further subgenotyped (Table 2) because only partial gene sequences or single preS, S, or C fragments were obtained. In all of the above strains, genotypes of the different fragments agreed with each other. In addition, 31 viruses were suspected mixed genotype infections or recombinants; they were not included in the above analysis and are discussed later.

**Table 2:** Prevalence of HBV genotypes and subgenotypes, excluding mixed or recombinant strains and untypeables, Haiti*

Genotype or subgenotype	No. (complete genomes; partial strains) of genotypeable or subgenotypeable strains	Genotypeable or subgenotypeable strains, %
A	128 (63; 65)	71.5
A1	77 (36; 41)	43.0
A2	1 (1; 0)	0.6
A5	35 (21; 14)	19.6
D	40 (5; 35)	22.4
D3	7 (2; 5)	3.9
D4	29 (3; 26)	16.2
E	11 (1; 10)	6.1
Total	179 (69; 110)	100

*HBV, hepatitis B virus.

### Phylogenetic Analysis

#### Subgenotype A1

**Figure A1:**
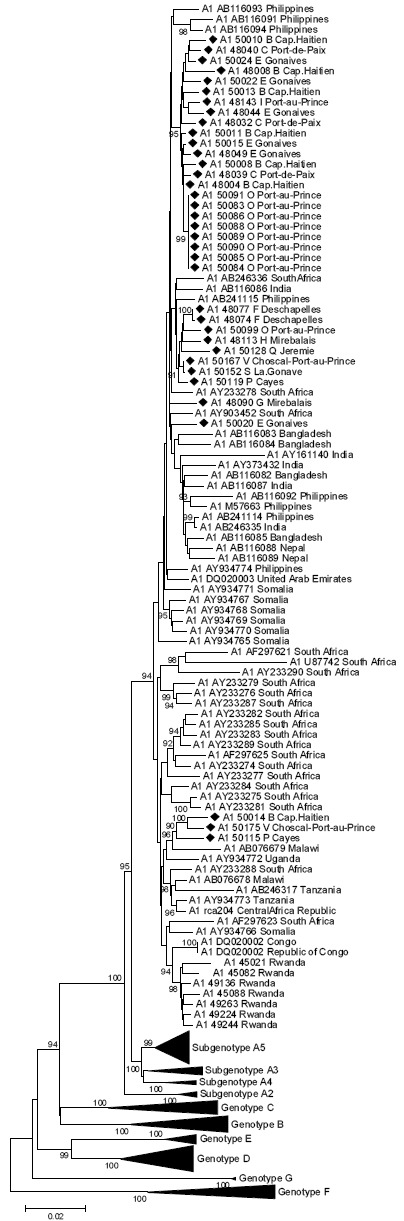
Phylogenetic analysis of selected sequences clustering with subgenotype A1, based on the complete genome. Diamonds indicate Haiti sequences. All complete A1 sequences available in GenBank are included.

Phylogenetic analysis of A1 complete genome sequences showed that Haiti strains form several clusters (not necessarily supported by bootstrap values) within available full-length A1 strains (Figure A1) from South Africa and other eastern African countries, as well as from the Philippines. Haiti’s complete genome A1 strains showed a mean genetic diversity of 1.45% (maximum diversity of 3.86%) that rose to a mean genetic diversity of 2.49% (maximum 6.61% between FJ692589 and U87742) when all available A1 strains (mean 2.87%, maximum 7.64% between AY161140 and U87742) were included.

#### Subgenotype A5

**Figure 1:**
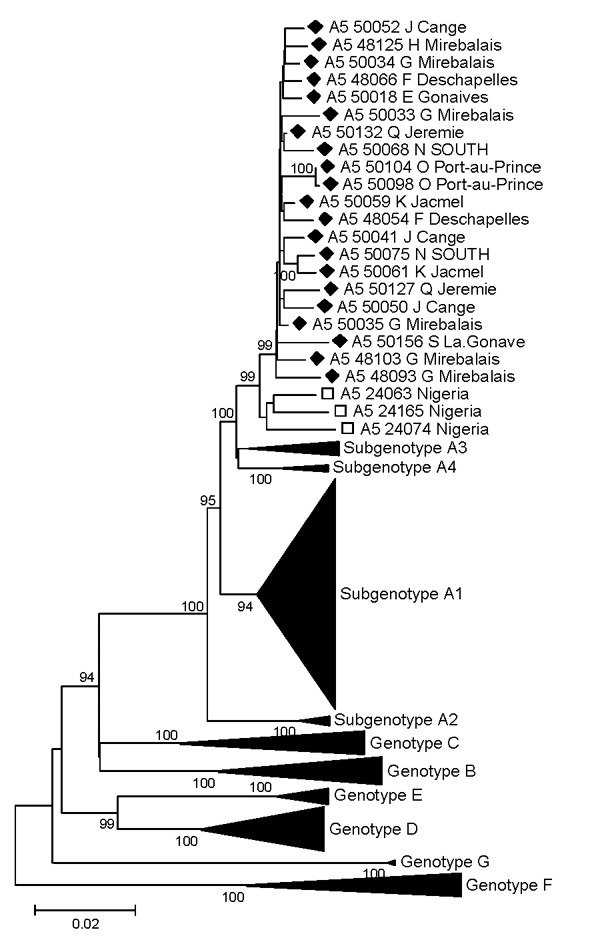
Phylogenetic analysis of selected sequences clustering with subgenotype A5, based on the complete genome. Diamonds indicate Haiti sequences; squares indicate Nigeria A5 strains. All complete A5 sequences available in GenBank are included. Scale bar indicates nucleotide substitutions per site.

A5 originally was proposed on the basis of the preS and preC/C gene fragments of 3 Nigerian strains (*20*). We present here the full-length sequences of the latter strains (accession nos. FJ692554–FJ692556) and compare them with all full-length A sequences from Haiti. Twenty-one sequences clustered with the only available A5 sequences from Nigeria. The overall mean intrasubgenotype diversity of A5 is 1.42% (maximum genetic diversity 2.89%). The mean intersubgenotype distance of the proposed A5 subgenotype was above the approximately 4% proposed for a new subgenotype (*3*,*22*) for subgenotypes A1 (4.1%), A2 (4.8%), and A3 (5.1%); it was 3.8% when compared with the previously proposed subgenotype A4 (*20*). Nevertheless, A5 strains from Haiti and Nigeria form 2 distinct phylogenetic subgroups within A5, supported by high bootstrap values (99%; Figure 1). These subgroups are separated by a mean, minimal, and maximum genetic distance of 2.28%, 1.71%, and 2.89%, respectively.

#### Genotype D

**Figure 2:**
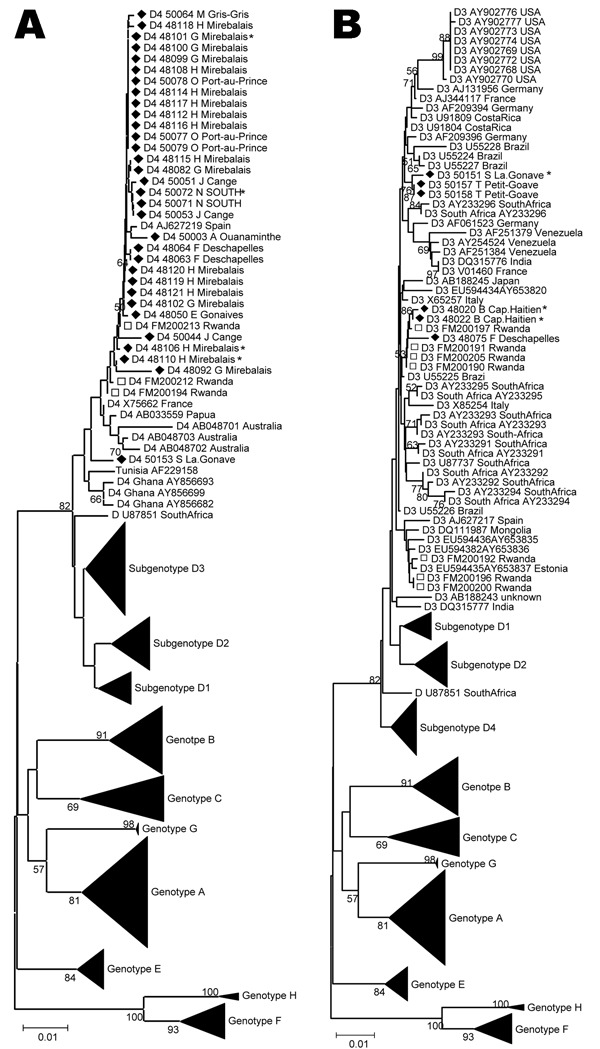
Phylogenetic analysis of selected sequences clustering with subgenotype D4 (A) or D3 (B), based on the S fragment, including potential mixed or recombinant strains (*). Diamonds indicate Haiti sequences; squares indicate Rwanda strains. Scale bar indicates nucleotide substitutions per site.

Because of low numbers of complete genome sequences available for genotype D in Haiti, we analyzed this genotype on the S fragment. In Haiti, most D strains belonged to D4 (29/179 [16.2%]). Besides a small cluster of D4 sequences from Australia and Papua New Guinea, a few D4 sequences from Rwanda (accession nos. FM200194, FM200212, and FM200213) (*9*) and single D4 sequences from Spain and France are available in GenBank. D4 sequences from Haiti were closely related to those from the latter 3 countries and somewhat separate from the Australian cluster (Figure 2, panel A). Despite the relatively high prevalence of D4 throughout most of Haiti, the mean genetic diversity of D4 S fragment sequences was only 0.39% (maximum diversity of 2.18%).

D3 (7/179, 3.9%) was less frequently found in Haiti than D4. Sequences seemed to form small geographic and genetic clusters, 1 of which most closely resembled strains from Rwanda (GenBank accession nos. FM200190, FM200191, FM200197, and FM200205) (*9*), but all D3 strains in Haiti were interspersed among strains from Brazil (Figure 2, panel B). Haiti D3 S fragment sequences showed a mean genetic diversity of 0.74% (maximum diversity of 1.44%).

#### Genotype E

**Figure A2:**
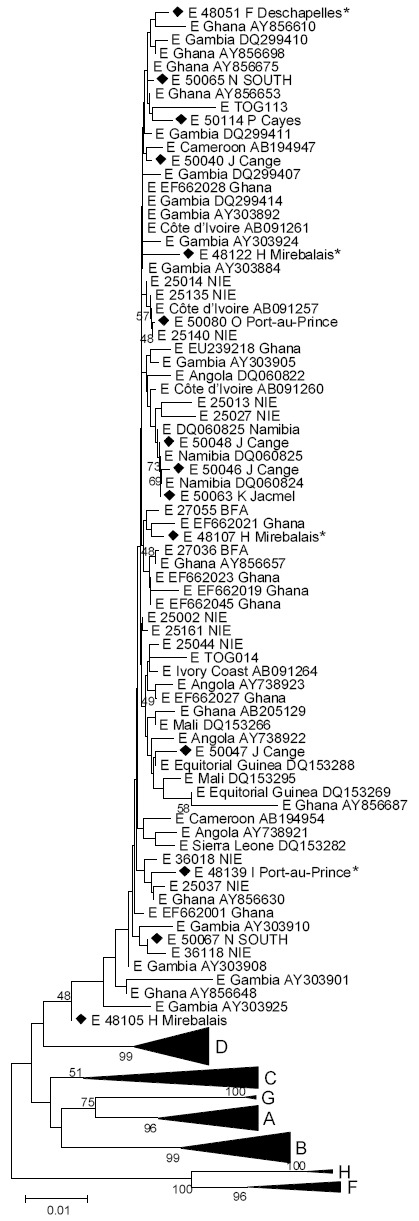
Phylogenetic analysis of selected sequences clustering with genotype E, based on the S fragment, including potential mixed or recombinant strains (*). Diamonds indicate Haiti sequences.

The prevalence of genotype E sequences was surprisingly low, with only 11 (6.1%) of the 179 subtypeable strains being classified as this genotype. Available S-fragment sequences clustered with African HBV/E strains and were interspersed as individual strains among those HBV/E strains (Figure A2). The mean and maximum genetic diversity of the Haiti S fragments were 0.76% and 1.93%, compared with 0.74% and 4.66% of all African genotype E strains.

#### Mixed Infections and Recombinations

We suspected mixed infections in 25 samples either because of at least 5 divergent nucleotides corresponding to discrepant (sub)genotypes in at least 1 overlapping region of the PCR fragments or because of divergent nucleotides within the fragments after additional PCR analyses**.** Mixed infections included all genotypes in Haiti, as well as 1 B4 (S-fragment) and 1 C (X-fragment) sequence. Six other strains showed possible recombinations within the preS fragment or the X fragment. PreS-fragment recombinants were based on HBV/E and HBV/A, whereas those in the X fragment emanated from HBV/G in recombination with genotypes D or A. One of these strains also showed signs of mixed infection. Although sequences were relatively short, recombination breakpoints seemed to be located around nucleotide 800 on the X gene; the location varied in the preS gene (nucleotide 330, 640, or 870).

## Discussion

More than 90% of today’s Haitian population is descended directly from African slaves (*19*) exported from the late 17th century through the early 19th century (*23*). Because vertical transmission and household transmission during early childhood are important routes of infection and are associated with excess risk for chronic disease, HBV is transmitted between generations (*24*,*25*). Thus, HBV strains in Haiti may to some extent reflect strains that were prevalent in Africa several centuries ago.

### Subgenotype A1

**Figure 3:**
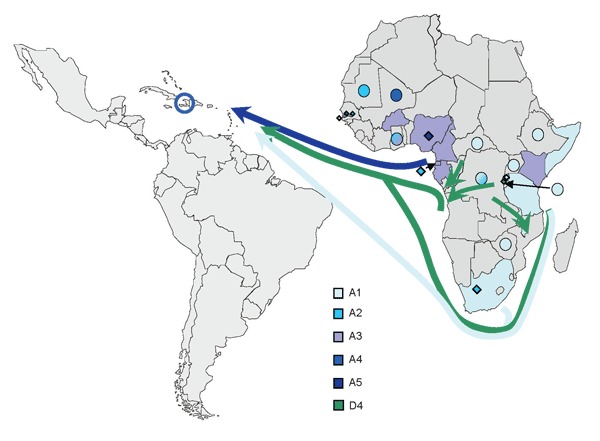
Distribution of hepatitis B virus A subgenotypes and D4 (only in Rwanda) in Africa and their potential routes of spread toward Haiti (color-coded arrows). Colored dots indicate African countries with <10 A strains available; full color indicates countries with >90% dominance of 1 subgenotype; or a 60%–90% predominance of 1 subgenotype, with minority subgenotypes shown as diamonds. Subgenotypes other than A1 and D4 are not shown for Rwanda. Sequences included were obtained from GenBank and unpublished data.

Forty-three percent of African HBV sequences belong to subgenotype A1, the main African A subgenotype. This subgenotype was found in most eastern African countries (*13*) and dominated in this region for which larger sets of HBV strains have been characterized, including Somalia, Kenya (*13*), Rwanda (*9*), and South Africa (*13*). Subgenotype A1 is essentially absent from West Africa (i.e., west of the African E/A1 genotype divide) (*9*) and from other continents. During the peak of Haiti’s slave importation during the late 18th century, almost 60% of captives came from southeastern and central Africa (*26*,*27*) (Figure 3). Complete Haitian A1 strains (36/77 A1 strains) formed several small clusters within A1, at least the largest of which is supported by a bootstrap value of 95%. Thus, the phylogeny is highly suggestive of multiple early introductions into Haiti of distinct A1 strains from eastern Africa that continued to spread in Haiti’s population.

### A5, a New Subgenotype

One third of HBV/A strains clustered with a group of rare strains that have been found only in southwestern Nigeria (*20*). Because only preS and preC/C sequences had been available, these strains had only provisionally been assigned to a new subgenotype, A5, until full-length sequences would become available. Complete A5 genome sequences from Haiti (n = 21) and Nigeria (n = 3) presented in this study showed a mean intrasubgenotype diversity of 1.42%. The mean intersubgenotype distances of A5 are above the approximately 4% of the definition of a subgenotype (*3*) for subgenotypes A1 (4.1%), A2 (4.8%), and A3 (5.1%). Compared with the proposed subgenotype A4 (3.8%), the mean intersubgenotype diversity is only slightly <4%. Thus, together with high bootstrap support (99%), these strains fulfill the formal definition, proposed by Kramvis et al. (*13*), of a new subgenotype A5.

A5 has been found only in southwestern Nigeria, the former Bight of Benin, where the first wave of slaves brought to Haiti originated (*27*). Interestingly, all A5 sequences from Haiti clustered together, but somewhat separately (bootstrap support of 99%) from those from Nigeria, suggesting early evolutionary separation of the 2 clusters. This distinct clustering further corroborates early introduction of these strains to Haiti during the slave trade. A5 strains in Haiti were considerably more homogenous than A1 strains, possibly reflecting their geographic confinement to and homogeneity in Africa.

Compared with subgenotype A5, A1 showed only a slightly higher mean genetic distance between African and Haitian strains (2.62% for A1; 2.28% for A5). However, when each Haitian A1 group is considered as a separate and independent introduction, the mean genetic diversity of these groups is only 0.8%–1.6% (for the different groups), indicating more recent introduction of these strains into Haiti.

### Genotype E

We have extensively investigated HBV genotypes in Nigeria (*18**,**20*; unpub. data). Of almost 300 sequences from both southern and northern Nigeria, 95% belong to genotype E and 4% to subgenotype A3. Thus, in Nigeria, the only location where HBV/A5 has been found, almost all HBV carriers are infected by genotype E, and the most prevalent none-E strains belong to subgenotype A3. In contrast, genotype A5 is rare and confined to Nigeria. Historical records (*27*) and the prevalence of A5 confirm that ancestors of the Haitian population came from the Bight of Benin, one of the most important slave trading posts. Nevertheless, only ≈6% of strains in Haiti belonged to genotype E. Single sequences were interspersed among current HBV/E sequences from Africa, with little genetic distance between them, which suggests that these HBV/E strains were introduced only recently into Haiti. The recent establishment HBV/E into Haiti strongly indicates that genotype E was essentially absent from West Africa when and where slaves were assembled for transport. Recent introduction of genotype E into the general West African population, as we have suggested previously (*18*,*20*), also would explain the low genetic diversity of this genotype.

### Subgenotype A3

A3, the minority subgenotype in West Africa, was virtually absent from Haiti, suggesting that this subgenotype also arrived later in the Bight of Benin. Interestingly, Cameroon is the only country where genotypes E and A3 cocirculate at similarly high prevalences (*18**,**28*), suggesting that both E and A3 may cooriginate from this region.

### Genotype D

More than 20% of sequences analyzed from Haiti belonged to genotype D (D3, D4). Subgenotype D4 strains are rare in the world, but we found a surprising 17% prevalence in Haiti. Interestingly, some of the D3 and the D4 strains were closely related to recent strains from Rwanda (*9*). With prevalences of 15.6% of D3 and 6.7% of D4 (*9*), Rwanda is also the only country where sizeable percentages of these 2 subgenotypes were found, further corroborating an origin of these strains from that part of Africa. During the second half of the 18th century, slaves were, to a large extent, collected in west-central Africa and shipped either from the western coast (*23*) or the eastern coast (*23*,*27*) to the Caribbean. Thus, an African origin of the D strains seems likely (Figure 3).

### Time of Evolution

Our results seem to agree with the time frame of the transatlantic slave trade. According to the simplest evolutionary model with a mutation rate of 4.2 × 10^–5^ (*29*), separation between Haitian and African A5 strains, with a mean genetic distance of 2.28%, would have occurred ≈270 years ago. When each Haitian A1 group is considered as a separate and independent introduction, the mean genetic diversity of 0.8%–1.6% corresponds to at least 100–190 years of evolution of each of the A1 groups in Haiti. These estimates for A1 and A5 seem to agree with the historical records that slaves (putatively infected with A5) from West Africa were introduced to Haiti ≈270 years ago, i.e., during the early phase (from the 1730s on) of the slave trade (*27*), and slaves (putatively infected with A1) from eastern Africa were exported to Haiti around the turn of the 18th century (*26*,*27*).

The apparent absence of old genotype E strains in Haiti indicates that it was rare in West Africa at the time of the slave trade and emerged in the general African population by the beginning of the 19th century, after the majority of the slave trade was suspended. Indeed, the mean genetic diversity (1.74%) would evolve over the complete genome in only ≈200 years, even from a single virus.

Given the recent introduction of genotype E, the excessively high prevalence of this genotype throughout the genotype E crescent is difficult to understand. If HBV antibody prevalence was as high at the time of the emergence of HBV/E as it is today, why did genotype E spread so much more efficiently than genotype A (subtype A3 or A5) in West Africa? No evidence exists to indicate that immunity to genotype A (e.g., through vaccines) does not protect against genotype E. The rare A/E recombinants do not suggest a long cocirculation of both genotypes at a high prevalence. A more likely scenario is that HBV was relatively rare in Africa until genotype E was massively spread by a new route of transmission. Much evidence points toward mass injection campaigns performed in the Belgian and French colonies at the turn of the 19th century. Treatment campaigns against yaws during the 1920s–1950s (*30*,*31*) and chemoprophylactic campaigns against sleeping sickness (*32*) were widespread and entailed sometimes reusing only a few syringes to treat, for example, 90,000 persons (*32*). Later, the extensive use of injectable antibiotics, vaccines, and other drugs (e.g., against syphilis) with unsafe needles further promoted HBV transmission (*30*,*31*). In Egypt, widespread transmission of hepatitis C virus has been linked to unsafe mass injection campaigns against schistosomiasis until the 1980s (*33*). Because HBV is estimated to be 10 times more transmissible than hepatitis C virus (*31*,*33*), injection with unsafe needles is a possible route of transmission of HBV (genotype E). In addition, early potentially contaminated vaccine preparations, as well as insect vectors, might be culpable in the spread of HBV infection.

The high prevalence of genotype A5 in Haiti strongly indicates that predecessors of the Haitian population came from the Bight of Benin. However, subgenotype A3 and genotype E, highly prevalent today in this part of Africa, are essentially absent in Haiti. This lack strongly indicates that HBV/E emerged only later in the general African population. The high prevalence of HBV/E in large parts of Africa further suggests that HBV hyperendemicity is a recent phenomenon and probably the result of the extensive use of unsafe needles.
